# Understanding the Evolution of Mammalian Brain Structures; the Need for a (New) Cerebrotype Approach

**DOI:** 10.3390/brainsci2020203

**Published:** 2012-05-18

**Authors:** Romain Willemet

**Affiliations:** 105 Chemin de la Salade Ponsan, Toulouse 31400, France; Email: r.willemet@gmx.com; Tel.: +33-760-265-906

**Keywords:** brain evolution, mammals, mosaic evolution, concerted evolution, cerebrotype, allometry

## Abstract

The mammalian brain varies in size by a factor of 100,000 and is composed of anatomically and functionally distinct structures. Theoretically, the manner in which brain composition can evolve is limited, ranging from highly modular (“mosaic evolution”) to coordinated changes in brain structure size (“concerted evolution”) or anything between these two extremes. There is a debate about the relative importance of these distinct evolutionary trends. It is shown here that the presence of taxa-specific allometric relationships between brain structures makes a taxa-specific approach obligatory. In some taxa, the evolution of the size of brain structures follows a unique, coordinated pattern, which, in addition to other characteristics at different anatomical levels, defines what has been called here a “taxon cerebrotype”. In other taxa, no clear pattern is found, reflecting heterogeneity of the species’ lifestyles. These results suggest that the evolution of brain size and composition depends on the complex interplay between selection pressures and constraints that have changed constantly during mammalian evolution. Therefore the variability in brain composition between species should not be considered as deviations from the normal, concerted mammalian trend, but in taxa and species-specific versions of the mammalian brain. Because it forms homogenous groups of species within this complex “space” of constraints and selection pressures, the cerebrotype approach developed here could constitute an adequate level of analysis for evo-devo studies, and by extension, for a wide range of disciplines related to brain evolution.

## 1. Introduction

From the tiny brain of the shrew, weighing less than one gram, to the huge brain of the sperm whale which weighs more than 8 kg, how have the major structures of the mammalian brain evolved? Because these structures are functionally distinct [1], one might expect that the brain of each mammal has evolved its own set of structure sizes that adapts it to its environment. However, given the complexity of the mammalian brain, from the formation of the neural tube to the high degree of connection between structures, one could suspect that the extent to which structure size can vary from one another should be limited. The understanding of the extent to which modularity and constraints act on brain design during evolution is a fundamental issue in neuroscience, because of the clues it gives to the organization of the brain [2] and in the understanding of differences in cognition and behavior in different species [3]. Theoretically, the manner in which brain composition can evolve is limited, ranging from highly modular (“mosaic evolution”) to coordinated changes of brain structure sizes (“concerted evolution”) or anything between these two extremes (see [1], p. 140). Remarkably, the two main theoretical frameworks that have emerged on this issue defend contrasting hypotheses regarding the relative importance of constraint and modularity. This is all the more puzzling since both hypotheses are based on the same mammalian dataset.

The first hypothesis, proposed by Finlay and Darlington [4] (and extended by [5]) assumes that because more than 96% of the variance in the size of brain structures is due to the size of the brain itself, the evolution of the mammalian brain can be defined as a “concerted evolution”, in which structures evolve together. Importantly, structures unevenly participate in the brain enlargement. For example, as brains get bigger, “the isocortex gets unusually large and the medulla stays unusually small” [5]. Following the concerted evolution theory, different brains are simply scaled up versions of one another (although with different, but predictable, proportions of each structure) because the unity of selection is the brain, instead of individual brain structures. The authors have proposed an evolutionary-developmental (evo-devo) basis for their results, called the “late equals large” model, in which developmental constraints limit the variability of the sizes of these structures. According to this model, changes in absolute brain size are the consequences of the stretching or squeezing of a conserved neurogenetic schedule, resulting in the concerted growth of all parts of the brain, structures developing the latest becoming the largest ones as brain size increases [4,5] (see also [6,7,8]).

Other authors have proposed a less constrained view of brain evolution, in which “mosaic evolution” plays a major role [9]. This theory differs from the “concerted evolution” theory developed by [4,5] for two reasons. First, it attributes the differences in the enlargement between structures to an artifact due to the presence of allometric grade shifts (reflecting taxonomic differences in the relative size of a brain structure), whereas authors of concerted evolution theory have repeatedly negated the importance of these grade shifts [5,10]. The second reason is that the structures that belong to a system (*i.e*., structures highly connected) tend to evolve in concert, independently from the other structures [9,11]. With the methodology used by Barton and Harvey [9], the same pattern seems to be shared by the two taxa studied (insectivores and primates). Hence, the selection appears to be able to directly act on structures or systems, instead of the entire brain. In fact, the importance of mosaic evolution seems to be so remarkable that it allows de Winter and Oxnard [12] to infer important ecological and behavioral attributes of a mammalian species (murinine bat) just by a multivariate analysis of the relative proportion of its brain structures. Furthermore, the mosaic selection of a brain structure is made possible by the heritability of structure size [13,14].

Given that both theories are often viewed as representing opposite ends of the spectrum, it has been stated that the debate is focused on their relative importance, more than favoring one over the other [1]. For example, Striedter has stated that “concerted evolution is a general principle that holds most of the time but not always” because “the great majority of species differences in region size do seem to fall well within the 2- to 3-fold limits [considered by Finlay and Darlington [4] as consistent with their hypothesis of concerted evolution]” ([1], p. 158, brackets added). In addition, Striedter has proposed that “mosaic evolution should be more common between classes than between orders, more common between orders than between families, and so forth” ([1], p. 158). This suggestion can be connected to the cerebrotype approach developed by Clark *et al*. [15]. Clark *et al*. were indeed the first to propose the term “cerebrotype” (“a species-by-species measure of brain composition” [15], see also [16]). They proposed that different mammalian taxa possess their own cerebrotype evolving by a succession of “shifts” due to mosaic evolution, and that, “within each taxon, brain regions are scalable, tending to maintain ﬁxed proportionality of size to one another independent of absolute total brain volume” [15]. Though the observation that mammalian species can be grouped by some aspects of their brain composition is of prime interest, one of the main propositions of the original approach (fixed proportions among taxa) should be revised, as highlighted in this present paper. Using multivariate analysis, de Winter and Oxnard [12] showed that different mammalian orders have different directions of evolution of their brain structures. The authors suggest “that either such [developmental] constraints are less powerful than we assumed and have been overridden by new selective pressures in the different lineages, or that the developmental constraints have themselves evolved differently since the separations of the original stocks, or both” ([12], brackets added). Their paper has an important role in this debate by suggesting that brain evolution differs between orders. However, the method they used makes the precise analysis of their result difficult (although Brown [17] attempted such analysis), because species positions on the PCA plan should depend on the sample of species included. 

Other researchers working on different vertebrate taxa have studied this issue, providing new insights. Iwaniuk *et al*. [18] suggested a major role of mosaic selection in the evolution of the avian brain. However, their study mixes different avian orders, and different “cerebrotypes” [19] following the meaning given by [15], so it is hard to know the part of their results due to possible grade shifts between cerebrotypes. Recently, Gonzalez-Voyer *et al*. [20] studied the relative influence of constraints and modularity in the evolution of cichlids brains. Using multivariate and sophisticated evolutionary analysis, their results suggest a predominant role for mosaic evolution in the determination of brain composition in cichlids. The mosaic evolution theory finds further support in fishes, with the presence of striking cases of mosaic evolution in teleosts and chondrichtyans (see [1], p. 154) or even inter-population differences in brain structure size [21]. However, since 86% of the variance in brain structure volume is explained by the brain size in [20], one cannot exclude that brain structures in cichlids evolve, at least to some extent, concertedly. 

Thus, most papers support a relatively large influence of mosaic evolution in the determination of brain composition in vertebrates, although a concerted pattern of the evolution of brain structure sizes is more or less found in all the taxa studied and cannot be ignored (see also [22]). Furthermore, a growing number of papers highlighting taxa-specific characteristics of the brain (see Discussion) must be added to these results at structure size level, which further complicates the issue. Thus, up until now, a comprehensive framework on how constraints and modularity have shaped the mammalian brain is still lacking.

In this paper, the data on mammalian brain structures sizes from Stephan, Baron and Frahm’s group [23,24], including the unpublished data of dozens of bat species from the same research group as well as recent data from Reep *et al*. [10] (the final dataset contains 376 mammalian species) are re-analyzed. This paper corroborates and extends other papers presenting taxa specific patterns of brain evolution [9,12,15] by defining groups of species with homogenous properties at many anatomical levels (“taxon cerebrotype” see also [15]). The mechanisms at the origin of these taxon cerebrotypes are discussed. Under the limits imposed by developmental and functional constraints, the evolution of brain composition (and in particular here, the size of brain structures) results from selection toward a particular composition, explaining both the allometric pattern between structures seen in a cerebrotype and the species-specific deviations. Within this view, the interplay between the constraints and selective pressures is assumed to be specific to each taxon cerebrotype. It is concluded that understanding the evolution of mammalian brain structures needs the integration of results from all anatomical levels of the brain in particular groups of species (taxon cerebrotypes). While applied here to the issue of brain structure evolution, the cerebrotype approach constitutes a valuable tool for a wide range of disciplines related to brain evolution. 

## 2. Results

### 2.1. Taxa-Specific Evolution of Brain Structure Sizes

**Figure 1:**
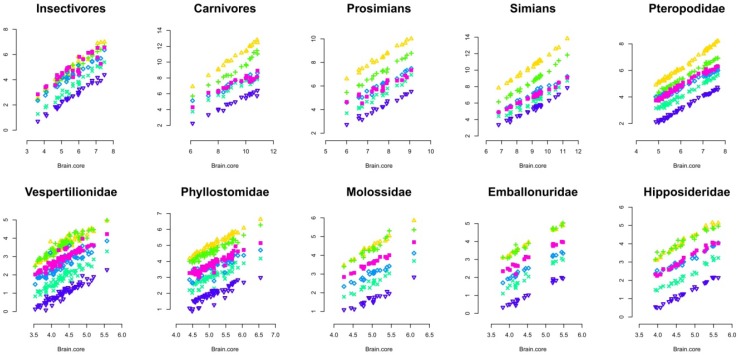
Regression of brain structure sizes onto brain core size (log scale). Taxonomic levels differ among taxa. Prosimians and simians (primates) are represented separately for reasons explained in the discussion. Due to the large number of bats species for which measurements are available, it is possible to present a detailed analysis for six bat families here, Pteropodidae, Vespertilionidae, Phyllostomidae, Molossidae, Emballonuridae, Hipposideridae. Legend: yellow triangle: neocortex; green plus: cerebellum; violet square: paleocortex; light blue lozenge: hippocampus; turquoise cross: schizocortex; blue triangle: septum.

Using all of the species of this dataset, the first component of the principal component analysis of all the 11 structure sizes (logarithms) explains 95% of the total variance, a percentage similar to that reported previously [4,5], using a smaller number of species. Moreover, the loadings of the principal components also correspond to those reported in these studies, with a first component highly loaded on all structures but the olfactory bulb, and a second factor mainly loaded on the olfactory bulb (results not shown here). Thus, most of brain structure size variation is explained by brain size alone, a result, which, at first, supports the concerted view of brain evolution. However, Barton and Harvey [9] have shown that this apparent homogeneity hides the presence of grade shifts. Moreover, Stephan and Pirlot [25] have found inter-family differences in the scaling of brain structure sizes in bats (though with a small data set), and several papers have suggested differences between mammalian orders in the correlation pattern between brain structures [12,26,27]. This raises the question of the extent to which we can consider the mammalian taxon as homogenous (Figure 1). The use of a non limbic brain core, as defined by [5] (the sum of the medulla, mesencephalon, diencephalon, and striatum sizes), has been preferred over other methods for comparison with previous studies [5,10].

Table 1 presents a definitive argument in favor of the necessity to analyze the evolution of the mammalian brain at the taxon level. In this analysis, the methodology used by Reep *et al*. [10] consisting of regressions of brain structure size onto the size of the non-limbic brain core as defined above has been applied. The regression slopes significantly differ between taxa in a consistent way, although not in every structures in the present dataset. This suggests that brain regions enlarge at different rates between taxa. Furthermore, taxa also differ in the relative size of a brain structure for a given brain size (allometric grade shifts [1,9]). Thus, we have to consider each taxon separately in the analysis of brain evolution, because there are no universal allometric rules for the evolution of brain structure sizes in mammals. Table 1 also indicates significant differences between the slopes of the structures inside taxa for seven out of ten taxa tested. In these taxa, the fact that these slopes significantly differ from isometry indicates that the relative sizes of brain structures vary with brain size. The following part describes these variations within and between taxa.

**Table 1:** Estimate of the slope (Major Axis Regression forced at the origin, see Methods) of phylogenetically independent contrasts (PIC) of the logged brain structure sizes regressed onto the independent contrast of the logged brain core. See Methods for a description of the test for common slope. Symbols for significance level are: *** *p* < 0.001, ** *p* < 0.01, * *p* < 0.05, NS *p* > 0.05.

	**Taxa (number of PIC)**	**Neocortex**	**Cerebellum**	**Schizocortex**	**Hippocampus**	**Septum**	**Paleocortex**	**Tests (LR)**
	**Insectivores (27)**	**1.138** (1.057–1.226)	**1.090** (1.009–1.179)	**1.095** (1.010–1.188)	**0.895** (0.810–0.987)	**0.878** (0.807–0.954)	**0.975** (0.824–1.151)	30.591 (***)
	**Carnivores (17)**	**1.297 **(1.205–1.399)	**1.152** (1.008–1.320)	**1.021** (0.781–1.336)	**0.936** (0.761–1.149)	**0.918** (0.740–1.135)	**0.973** (0.811–1.166)	21.076 (***)
	**Prosimians (15)**	**1.121** (1.045–1.203)	**1.081 **(1.021–1.144)	**1.054** (0.936–1.187)	**0.975** (0.874–1.087)	**0.924 **(0.853–1.001)	**0.838** (0.701–0.997)	20.525 (***)
	**Simians (25)**	**1.315** (1.235–1.412)	**1.285** (1.196–1.381)	**0.990** (0.836–1.174)	**0.977** (0.840–1.136)	**1.008** (0.886–1.146)	**0.952** (0.840–1.079)	39.988 (***)
**Chiroptera**	**Pteropodidae (40)**	**1.101** (1.057–1.148)	**0.991** (0.941–1.044)	**0.919** (0.852–0.991)	**0.840** (0.768–0.918)	**0.965** (0.920–1.013)	**0.956** (0.904–1.012)	38.297 (***)
	**Vespertilionidae (58)**	**1.169** (1.076–1.272)	**1.140 **(1.052–1.235)	**1.223 **(1.099–1.364)	**1.327** (1.150–1.541)	**1.104** (1.017–1.199)	**1.094** (0.998–1.200)	**7.463 (NS)**
	**Phyllostomidae (50)**	**1.138** (1.063–1.220)	**1.078 **(1.006–1.157)	**1.109** (0.985–1.250)	**1.065** (0.918–1.237)	**1.109** (0.988–1.246)	**1.163** (1.035–1.309)	**2.165 (NS)**
	**Molossidae (16)**	**1.291** (1.191–1.402)	**1.182** (1.000–1.402)	**1.090** (0.951–1.250)	**1.003** (0.884–1.138)	**0.929** (0.770–1.118)	**1.011** (0.931–1.097)	21.16 (***)
	**Emballonuridae (16)**	**1.163** (1.070–1.267)	**1.182** (1.008–1.390)	**1.321** (1.091–1.617)	**1.057** (0.946–1.182)	**1.104** (0.935–1.307)	**1.220 **(0.972–1.549)	**5.237 (NS)**
	**Hipposideridae (19)**	**1.291** (1.225–1.361)	**1.065** (0.940–1.207)	**1.144** (1.036–1.266)	**1.006** (0.919–1.103)	**1.057** (0.990–1.128)	**1.152 **(1.072–1.238)	27.83 (***)
**Tests (LR)**	**All taxa**	44.038 (***)	31.586 (***)	27.495 (***)	33.349 (***)	28.451 (***)	30.516 (***)	
	**All taxa minus Chiroptera**	16.603 (***)	14.169 (**)	**1.388 (NS)**	**1.779 (NS)**	**3.320 (NS)**	**2.210 (NS)**	
	**Chiroptera Taxa**	24.791 (***)	13.266 (*)	25.751 (***)	28.619 (***)	13.734 (*)	22.263 (***)	

### 2.2. Brain Structure Evolution in Mammalian Taxa

**Figure 2:**
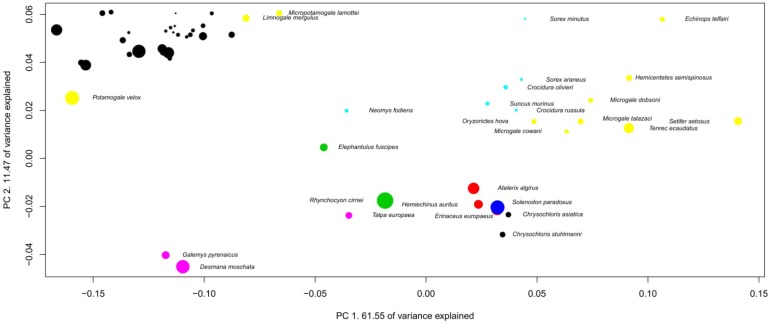
Phylogenetic Principal Component Analysis (PPCA) of insectivores’ brain structure proportions. Regression on independent contrasts of logged absolute brain size onto independent contrasts of position on PC1: Multiple *R*-squared: 0.067, *F*-statistic: 1.870 on 1 and 26 DF, *P* = 0.1832; PC2: Multiple *R*-squared: 0.289, *F*-statistic: 10.58 on 1 and 26 DF, *P* = 0.003156. Families are: black: Chrysochloridae, red: Erinaceidae, green: Macroscelididae, dark blue: Solenodontidae, light blue: Soricidae, purple: Talpidae, yellow: Tenrecidae.

**Figure 3:**
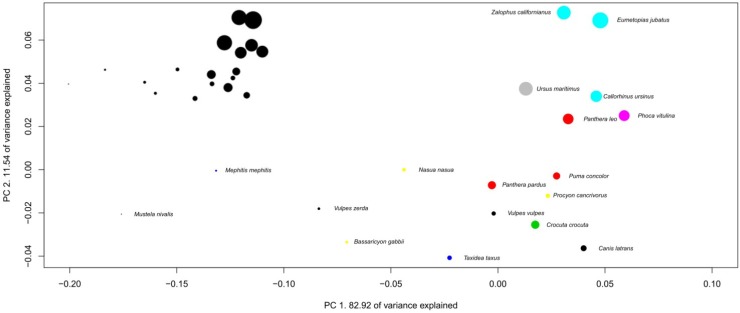
Phylogenetic Principal Component Analysis of carnivores’ brain structure proportions. Regression on independent contrasts of logged absolute brain size onto independent contrasts of position on PC1: Multiple *R*-squared: 0.791, *F*-statistic: 60.63 on 1 and 16 DF, *P* = 7.858 × 10^−7^; PC2: Multiple *R*-squared: 0.077, *F*-statistic: 1.332 on 1 and 16 DF, *P* = 0.2654. Families are: black: Canidae, red: Felidae, green: Hyaenidae, dark blue: Mustelidae, light blue: Otariidae, purple: Phocidae, yellow: Procyonidae, grey: Ursidae.

**Figure 4:**
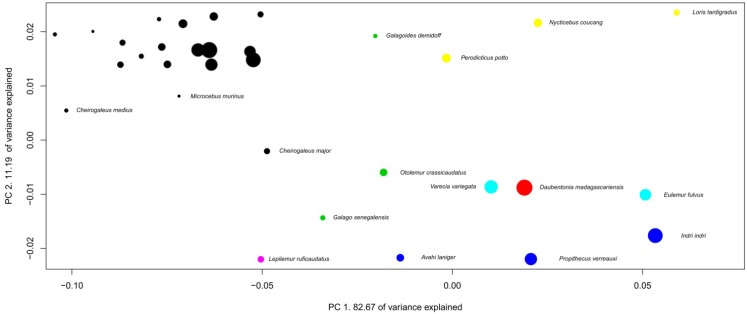
Phylogenetic Principal Component Analysis of prosimians’ brain structure proportions. Regression on independent contrasts of logged absolute brain size onto independent contrasts of position on PC1: Multiple *R*-squared: 0.489, *F*-statistic: 13.39 on 1 and 14 DF, *P* = 0.002575; PC2: Multiple *R*-squared: 0.143, *F*-statistic: 2.327 on 1 and 14 DF, *P* = 0.1494. Families are: black: Cheirogaleidae, red: Daubentoniidae, green: Galagidae, light blue: Lemuridae, dark blue: Indriidae, purple: Lepilemuridae, yellow: Lorisidae.

**Figure 5:**
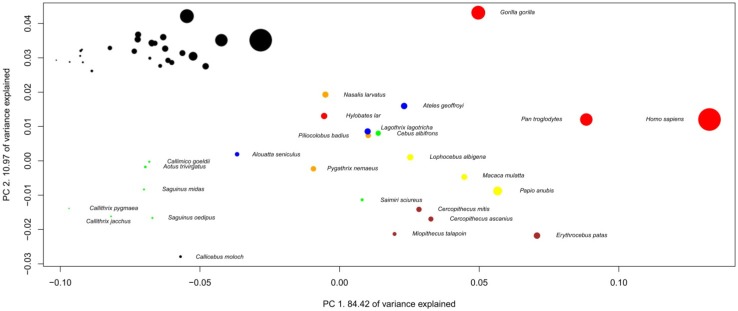
Phylogenetic Principal Component Analysis of simians’ brain structure proportions. Regression on independent contrasts of logged absolute brain size onto independent contrasts of position on PC1: Multiple *R*-squared: 0.706, *F*-statistic: 57.72 on 1 and 24 DF, *P* = 7.771 × 10^−8^; PC2: Multiple *R*-squared: 0.132, *F*-statistic: 3.655 on 1 and 24 DF, *P* = 0.0679. Families are: black: Pitheciidae, red: Hominoidea, green: Cebidae, blue: Atelidae, brown: Cercopithecini, orange: Colobinae, yellow: Papionini.

**Figure 6:**
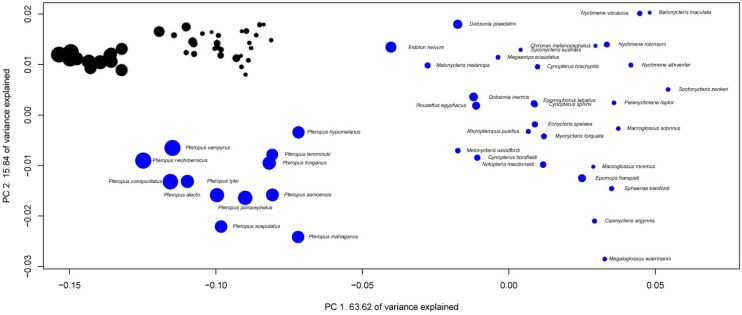
Phylogenetic Principal Component Analysis of Pteropodidaes’ brain structure proportions. Regression on independent contrasts of logged absolute brain size onto independent contrasts of position on PC1: Multiple *R*-squared: 0.485, *F*-statistic: 36.73 on 1 and 39 DF, *P* = 4.272 × 10^−7^; PC2: Multiple *R*-squared: 0.0036, *F*-statistic: 0.1427 on 1 and 39 DF, *P* = 0.7077.

**Figure 7:**
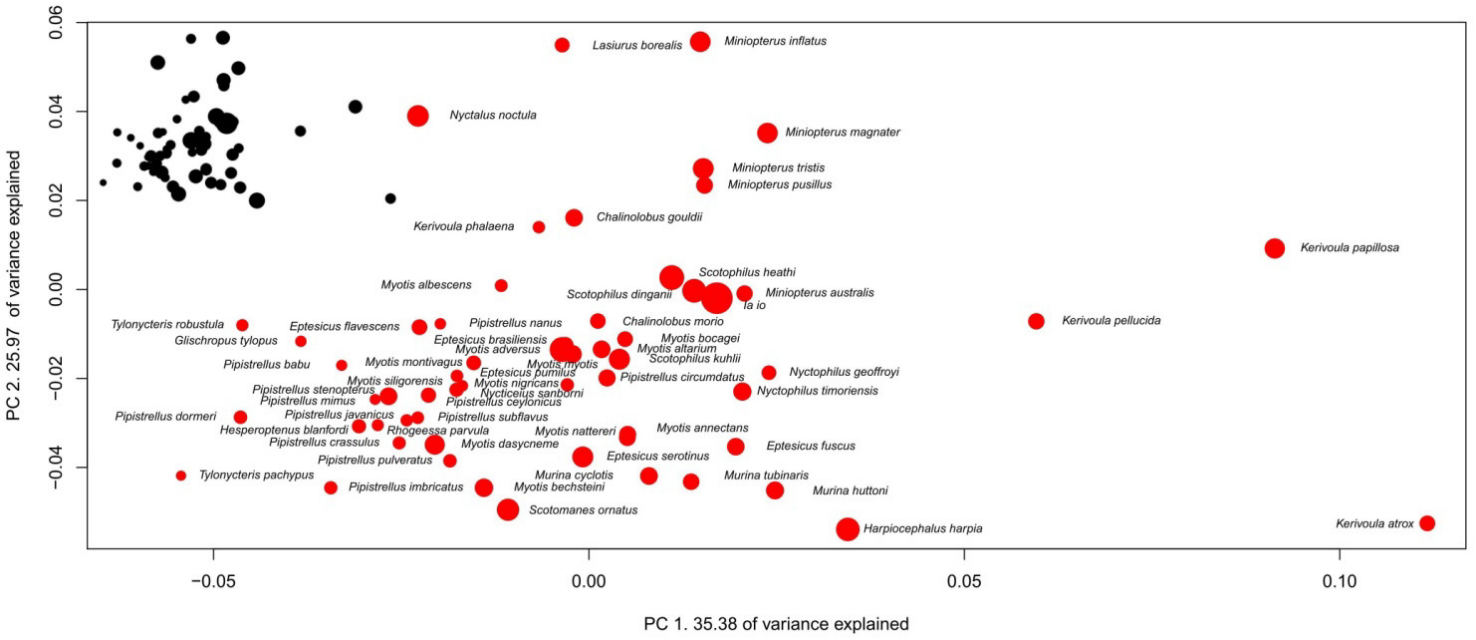
Phylogenetic Principal Component Analysis of Vespertilionidaes’ brain structure proportions. Regression on independent contrasts of logged absolute brain size onto independent contrasts of position on PC1: Multiple *R*-squared: 0.160, *F*-statistic: 10.85 on 1 and 57 DF, *P* = 0.001702; PC2: Multiple *R*-squared: 0.0047, *F*-statistic: 0.2666 on 1 and 57 DF, *P* = 0.6076.

The preceding section suggests that mammalian brains evolve in a characteristic manner between taxa. This section further examines this issue by using multivariate analyses. Several variables can be used in multivariate analysis for examining brains of different species. Using absolute volume can provide an insight into the allometric relationships between brain structures (see [20]), as can using absolute volume in regression analysis (Figure 1 and Table 1). The use of relative proportions of brain structures (see [12]) can be particularly appropriate in analyzing precise specific adaptations in brain structure size, because it considers structure sizes independently of brain size. Finally, using absolute proportions is an efficient way to characterize species brain composition [15]. Each method is useful and possesses its own advantages, and we can expect a lot when these three methods are used simultaneously in future detailed analyses of cerebrotypes. Using absolute proportions has the advantage of producing results directly comparable with those from Clark *et al*. [15]. The following analysis represents the evolution of the brains inside taxa by using three dimensions phylogenetic principal component analysis on brain structure proportions (3D Phylogenetic Principal Component Analyses (PPCA), Figure 2, Figure 3, Figure 4, Figure 5, Figure 6, Figure 7, Table 2, Supplementary Figure S1–S4 and Table S1). The variations of the slopes observed in Table 1 suggest that in most of the taxa, the relative size of each structure could vary with brain size. For this reason, correlations between positions of the species on the two firsts principal components have been calculated and are indicated in the figures legends. Also, in order to graphically represent the evolution of brain composition in the different taxa, a third dimension (symbol size) representing species’ relative brain size compared to the other species has been added to the graphs (see Methods).

Species positions on PPCA components depend on their brain composition (their brain structure proportions), so that the distance between species reflects differences in brain composition. Two factors, however, must be taken into account when interpreting the PPCAs presented above. First, the range of brain sizes inside taxa is expected to have an impact on the allure of the figure, since the smallest the range of brain size variation inside a taxon, the largest the impact of small scales variations (species-specific adaptations, see below). Second, in order to clearly represent the position of all the species, the graph properties used here do not represent the real scale of the PPCA; that is, *y* and *x* axes have different scales. The graph at the real scale is represented at the top left of each PPCA graph. In all cases, uncertainties in the phylogenetic relationships among the species in most of the taxa presented here (see Methods) prevent for a detailed analysis of species position. However, the patterns formed by the species on the component space are already highly informative. In fact, what these analyses suggest is that the evolution of the brain structure sizes inside each taxon differs in two aspects; the homogeneity and the direction of evolution. 

The homogeneity of brain evolution inside a taxon refers to the predictability of a species brain composition, based on the composition of the brain of the other species of the taxon. In the analysis at the absolute size level (regressions Figure 1), the homogeneity of the taxon is determined by high coefficients of determination (*R*^2^ not shown here). In the PPCA, a high degree of homogeneity appears with a high percentage of variance explained by the first principal component, the fact that most structures have high loadings on the first principal components, as well as the high correlation between brain size and positions on the first principal components. As indicated in the legends of the 3D PPCAs, positions of species on PC1 or PC2 are significantly correlated with their brain sizes in most of the taxa. In some taxa (insectivores and Vespertilionidae for example), one can observe a diversity of brain composition, even in phylogenetically close species (although insectivores probably is a polyphyletic taxon [28]) and between species with similar brain sizes (Figure 2 and Figure 7). The main factor at the origin of this variability could be the heterogeneity of ecological conditions between species belonging to these taxa, since it has been observed that vertebrate brain composition depends both on phylogeny and ecology (mammals [12,15,17]; birds [19,29]; teleosts [30,31]; chondrichtyans [32,33]). On the contrary, the simian’s taxon (as well as the Pteropodidae’s and carnivores’ taxa for example, Figure 3, Figure 5 and Figure 6) possesses a particularly homogenous schema of brain evolution (Figure 1). Results presented above indicate that brains inside homogenous taxa represent the variations of a certain schema of brain. In these taxa, the pattern of changes of the brain structure sizes is so persuasive that it gives a concerted pattern of evolution when the brains are sorting by their sizes. In this paper, the term “taxon cerebrotype” is used to label the pattern of brain evolution seen in such homogenous taxon. Contrary to what the original definition of “taxon cerebrotype” implies (see [15]), Table 1 and the correlations between absolute brain size and the positions on the component space show that brain composition evolves with brain size within a taxon cerebrotype. For this reason, the label “taxon cerebrotype” refers here to the brain composition and its evolution inside a homogenous taxon (taxon for which the evolution of brain structure sizes follows a concerted pattern, see above). This definition is extended in the Discussion.

**Table 2:** Loadings of the brain structures for the five first principal components of PPCA for each taxon of the Figure 2 to Figure 7. Scores higher than 0.5 are highlighted in grey.

	**PC 1**	**PC 2**	**PC 3**	**PC 4**	**PC 5**		**PC 1**	**PC 2**	**PC 3**	**PC 4**	**PC 5**	
**Insectivores**	**61.550**	**11.472**	**10.156**	**8.995**	**3.957**	**% Variance**	**82.921**	**11.539**	**3.039**	**1.098**	**0.744**	**Carnivores**
	−0.505	0.714	−0.380	−0.173	0.247	**Medulla**	−0.869	−0.040	−0.185	0.059	0.436	
	−0.434	−0.177	−0.387	0.790	−0.022	**Cerebellum**	−0.510	0.859	0.018	0.001	−0.028	
	−0.118	0.456	0.268	−0.314	−0.615	**Mesencephalon**	−0.870	−0.246	−0.168	0.293	−0.032	
	−0.290	0.084	0.210	−0.340	−0.600	**Diencephalon**	−0.702	−0.534	−0.009	0.410	−0.018	
	0.031	−0.022	0.190	0.180	−0.058	**Striatum**	−0.551	−0.411	0.652	0.080	−0.262	
	0.211	0.447	0.169	−0.026	−0.073	**Septum**	−0.752	−0.212	−0.258	0.155	−0.083	
	0.961	−0.174	−0.027	−0.105	0.170	**Paleocortex**	−0.647	−0.344	0.449	−0.458	0.110	
	0.107	0.210	0.880	0.331	0.213	**Hippocampus**	−0.561	−0.312	−0.712	−0.201	−0.193	
	−0.077	−0.238	0.083	−0.089	−0.414	**Schizocortex**	−0.490	−0.616	0.233	−0.240	0.057	
	−0.908	−0.354	0.012	−0.200	0.089	**Neocortex**	0.999	0.037	−0.005	0.003	0.002	
	0.922	−0.151	−0.280	−0.080	−0.103	**Olfactory bulb**						
	**PC 1**	**PC 2**	**PC 3**	**PC 4**	**PC 5**		**PC 1**	**PC 2**	**PC 3**	**PC 4**	**PC 5**	
**Prosimians**	**82.668**	**11.193**	**2.656**	**1.501**	**1.091**	**% Variance**	**84.421**	**10.966**	**2.323**	**1.036**	**0.544**	**Simians**
	−0.933	−0.122	−0.023	−0.029	−0.093	**Medulla**	−0.845	−0.315	0.235	−0.085	0.163	
	−0.167	−0.908	0.379	−0.011	−0.034	**Cerebellum**	−0.285	0.958	0.023	0.006	0.028	
	−0.801	0.112	−0.094	0.526	−0.211	**Mesencephalon**	−0.758	−0.422	0.336	0.246	−0.175	
	−0.143	−0.408	−0.755	−0.098	−0.380	**Diencephalon**	−0.867	−0.348	−0.113	0.211	0.234	
	0.367	0.273	−0.133	−0.779	−0.316	**Striatum**	−0.529	−0.073	−0.829	0.030	−0.132	
	−0.890	0.314	−0.066	0.010	0.087	**Septum**	−0.617	−0.366	0.247	0.062	−0.096	
	−0.621	0.755	0.197	−0.022	0.009	**Paleocortex**	−0.763	−0.259	0.330	0.275	−0.286	
	−0.837	−0.258	−0.376	0.007	0.277	**Hippocampus**	−0.769	−0.369	−0.032	−0.493	0.051	
	−0.370	−0.449	−0.103	−0.032	0.741	**Schizocortex**	−0.689	−0.168	0.365	−0.332	−0.252	
	0.999	0.032	0.003	0.019	0.006	**Neocortex**	1.000	−0.011	−0.002	0.003	0.009	
	−0.718	0.601	0.288	0.006	0.091	**Olfactory bulb**	−0.491	−0.111	0.253	−0.100	−0.305	
	**PC 1**	**PC 2**	**PC 3**	**PC 4**	**PC 5**		**PC 1**	**PC 2**	**PC 3**	**PC 4**	**PC 5**	
**Pteropodidae**	**63.620**	**15.838**	**9.076**	**5.546**	**2.789**	**% Variance**	**35.384**	**25.968**	**18.225**	**13.012**	**2.668**	**Vespertilionidae**
	0.769	−0.254	0.205	0.044	0.438	**Medulla**	−0.802	−0.003	−0.290	−0.444	−0.227	
	0.412	−0.795	0.360	0.105	−0.238	**Cerebellum**	0.130	0.968	−0.146	0.151	−0.010	
	0.597	−0.136	−0.084	0.523	0.390	**Mesencephalon**	−0.703	0.078	0.274	−0.514	0.273	
	−0.077	0.269	0.347	0.527	0.510	**Diencephalon**	−0.542	−0.425	0.008	−0.293	−0.108	
	−0.592	0.177	0.265	−0.563	−0.065	**Striatum**	−0.109	−0.117	0.503	0.104	0.619	
	0.407	−0.080	−0.006	−0.234	0.323	**Septum**	−0.286	−0.530	−0.321	0.382	0.129	
	0.375	0.417	0.370	−0.682	0.047	**Paleocortex**	−0.411	−0.395	0.350	0.705	−0.055	
	0.757	−0.042	−0.637	−0.055	−0.083	**Hippocampus**	0.624	−0.364	−0.684	0.007	0.030	
	0.401	−0.284	−0.671	−0.160	−0.086	**Schizocortex**	0.458	−0.338	−0.281	−0.023	0.286	
	−0.994	−0.063	−0.081	0.018	−0.016	**Neocortex**	0.805	−0.020	0.520	−0.260	−0.112	
	0.177	0.879	0.164	0.310	−0.266	**Olfactory bulb**	−0.290	−0.266	0.135	0.752	−0.273	

The direction of brain evolution inside a cerebrotype corresponds to the differential enlargement of brain structures. Importantly, only homogenous taxa (for which there are clear taxon cerebrotypes) can be analyzed this way. This is so because the species-specific variability in heterogeneous taxa is very high, so that the meaning of the allometric relationships between structures could be different than in cerebrotypes (in which species fit closely to the concerted pattern). The phylogenetic level of the taxon matters, in a way that even in the highly heterogenous Vespertilionidae taxon for example, one can find some clear subfamily or genus cerebrotypes. For sake of simplicity, however, only taxa that appear homogenous considering the phylogenetic levels described in Figure 1 are considered here. Inside these cerebrotypes, structures evolve concertedly. PCA loadings (Table 2) indicate that some structures (variable among taxa) explain most of the variance in the data, thereby predominantly influencing species positions on the component space. As a whole, these results indicate that the role of each structure in the brain enlargement differs between taxa. A detailed analysis of this topic is beyond the scope of this paper, and further studies should examine how these results correlate with differences in the ecology of these taxa. Importantly, although the terms “enlargement” or “evolution” used in this paper imply a direction of evolution from small brains to big ones, the cerebrotype approach developed here is compatible with a bidirectional evolution of the size of the brain during evolution [34,35].

## 3. Discussion

### 3.1. Defining a “Taxon Cerebrotype”

The original definition of taxon cerebrotype did not consider the evolution of brain composition inside a taxon [15]. This explains some of the criticisms that the concept received. For example, Barton comments that “this conflation of brain size and size-independent structural differences limits the utility of the cerebrotype measure for evaluating adaptive patterns and phylogenetic relationships. For example, cerebrotypes place gibbons (*Hylobates*) with similarly sized Old World monkeys, and not with their closest phylogenetic relatives, the other apes” [36]. The proposal presented here indicates that the place of gibbons inside the simian’s cerebrotype is mainly due to the size of their brains, which places them near simians’ species with similar brain size (and not necessarily with similar body size, see also [37] for a similar observation at the neuronal level). Intuitively, however, the concept of taxon cerebrotype, following the meaning of this present paper, has existed for a long time, since researchers on “human specializations” are looking at deviations of the pattern found in primate species, and not in random mammalian species, to study human brain evolution (see [38] for an example).

Actually, recent findings indicate that cerebrotypes represent a set of characteristics not limited to the size of the major brain structures. For example, Pillay and Manger [39] have shown that the patterns of girification of cerebral cortex vary among orders, in a way that each order has its own allometric pattern which significantly differs from the other orders examined (rodents, primates, ungulates and carnivores). On a smaller scale, Bush and Allman [40] show that different scaling rules apply for the size of the frontal cortex in primates and carnivores. Importantly, their results indicate that “the hyperscaling of primate frontal cortex is a regular and systematic relationship with size” [40], an observation consistent with the concerted evolution of brains inside cerebrotypes presented here. Also, for a given cortical size, the sizes of individual areas signiﬁcantly differ between taxa (marsupials, insectivores and primates) [41]. Moreover, there are differences in the cortical layering between orders (rodents, carnivores and primates) and similarities among species within an order [42], suggesting that cerebrotype characteristics affect virtually all levels of brain anatomy. Probably the most studied advances on this issue have resulted from the recent development of an easy method to count the number of neuronal and non-neuronal cells (the isotropic fractionator method [43]), that has made the study of neuronal scaling rules between taxa possible. Using this method, Herculano-Houzel and colleagues [44,45,46] have shown that there are different neuronal scaling rules between orders (rodents, insectivores and primates). Basically, their papers indicate that both the neocortex and the cerebellum have taxon-specific cell numbers (reviewed in [47]). This kind of research opens a new dimension in the study of brain evolution which, until now, completely matches the results raised in this study at the structural level. However, it must be noticed that, contrary to most literature on the subject [9,11,45,48,49,50], prosimians and the simians species have been analyzed separately, because of the possibility of two distinct cerebrotypes (see Table 1). For example, significant differences have been reported between these two taxa, in the size of the frontal cortex for a given cortex size [40], and the size of the primary visual cortex [51].

Thus, a taxon cerebrotype is a concept that encompasses many features that design the brains of a homogenous group of species, and further studies are needed to understand what best characterizes a cerebrotype, *i.e*., using molecular, genetic, cellular, developmental, behavioral and ecological markers that group species inside cerebrotypes. Indeed, using all mammalian species, one can separate the different taxa, based on their taxa-specific cerebrotypes, then different groups, and so on, until the separation of each species by their species cerebrotypes. In the absence of any objective markers, cerebrotypes should be defined when the grouping of species is ecologically and phylogeneticaly coherent, or, in practical term, when the separation of several groups becomes difficult, considering their allometric characteristics. Of course, depending on the goal of the study and the data available, such definitions should be adjusted. While Manger [52] suggests putting order at the system level in mammalian brain evolution, the presence of differences at the family level in Chiroptera, implies being more precise and to consider cerebrotype instead.

### 3.2. Toward a Global Framework

Probably the most important issue that affected the mosaic *versus* concerted evolution debate is a methodological one. Like any other allometric analysis, brain evolution studies are under the influence of the range of brain sizes included in the analysis. At the mammalian level, the concerted pattern created by the scaling of brain structure sizes is remarkable [1,4,5], and gives the impression that mammalian species are under the same rules of development (therefore supporting the concerted evolution hypothesis). When the range of brain size is reduced to the extreme (two brains of similar size), then differences in brain composition become evident. For example, there is a 5 fold difference in the size of the neocortex between insectivores and primates species for a non-cortical cortex of similar size [9] (therefore supporting the mosaic evolution hypothesis). Both levels raise interesting questions, but each of them taken individually gives an incomplete picture of this issue. Therefore the determination of the most relevant level of analysis is of fundamental importance. Which level permits to study both the developmental mechanisms that produce the brain, and the selective pressures that shape it?

#### 3.2.1. Evo-Devo Mechanisms

The presence of various taxon cerebrotypes, the diversity of brain composition in heterogeneous taxa as well as the presence of extreme cases of mosaic evolution [9,53] suggest that at least some of the developmental mechanisms controlling brain architecture in mammals have been continually under selection during mammalian evolution (see also [12]). In rodents for example, the superior colliculus of ground squirrels is around 10 times larger than in rats [53], whereas both species have similar brain and body size. Authors of the concerted evolution hypothesis originally stated that such outlier “does not negate the statistical description of the other data” [5]. This is true, since most of the variance in brain structure size is accounted for by the size of the brain itself ([1,4,5] and present study). However, the presence of outliers has important consequences on the biological interpretation of the data. Indeed, if ground squirrels have found the necessary tools to overcome potential developmental constraints and to grow such a large superior colliculus, then it is reasonable to suspect that these tools are available to other species.

Evo-devo studies have so far highlighted at least two fundamental aspects of the mechanisms at the origin of variation and constancy in brain. The first is that not everything is possible in brain evolution, that is, not all developmental mechanisms can be under selection. Indeed, a large part of the brain developmental mechanisms are conserved because they control the basic plan of the mammalian brain [54]. The second is that only a few mechanisms could be responsible for the diversity of changes seen in mammalian brains. Such mechanisms include alterations in neurogenesis timing and cell cycle rates as well as boundary shifts in brain patterning (reviewed in [55,56]). The relatively strong relationship between the allometric scaling of brain structures and neurogenesis observed by [4,5] suggest that interspecies differences in brain structure could mainly result from alterations of the neurogenetic schedule. Moreover, the model linking neurogenesis with brain structure allometry has continually evolved since its first formulation [4,7,57] and is progressively shifting toward a framework compatible with the cerebrotype approach developed here. For example, Clancy *et al*. [58] note that there is “a systematic deviation in the expected timing of neurogenesis in limbic and cortical structures for primates *versus* other mammals” that forces them to adjust the model for primate species [59]. As the number of data will rise, such taxa specific adjustments would probably be necessary to account for the approach presented here. In this context, the constant redefinition of the model and its implications (reviewed in [7]), as well as the continual remodeling of the open access database of neurodevelopmental events [60] are fundamental steps toward this goal.

#### 3.2.2. Selection and Constraints

Once the mechanisms governing the mammalian brain “model” have been settled in earlier mammalian species, selection has continually acted on the flexible developmental mechanisms controlling brain size and composition. As a result, there are fundamental differences between taxa regarding the relative size of brain structures ([9,12,15] and present study), the number and density of neuronal and nonneuronal cells inside brain structures (reviewed in [47]), the distribution and size of cortical areas [41,61] and the characteristics discussed in the first part of the Discussion (and probably many others). 

However, neurodevelopmental mechanisms often imply a succession of interdependent events [55,56] and some changes could be hard if not impossible to produce. At the structural level for example, it is likely that structures (for instance, layered structures or nuclei) respond differently to selection and constraints. In particular, the olfactory bulb seems to be under particularly loose developmental mechanisms [4,5,10,20]. In that case, some brain architectures could be more easily achieved than others. In addition to these developmental constraints, functional constraints further limit the variability of possible brain patterns [62]. To further complicate this issue, since different taxa have developed different brain architectures (at various anatomical levels), these constraints could differ between taxa. Therefore, the multitude of taxon cerebrotypes should not be seen as variations around a mammalian trend, as such trend apparently does not exist. Instead, each taxon, or more exactly, each cerebrotype, should be considered as a particular entity of brain evolution (related by their evolutionary history).

Following this view, when species of a homogeneous taxon (cerebrotype) occupy different positions of their taxon-specific ecological niche, the interplay between shared (among species) directions of selective pressures and constraints (both developmental and functional) produces the concerted evolution of brain structures (for example Figure 3, Figure 5 and Figure 6). This implies that, a priori, it is impossible to determine whether a coordinated pattern between two characters is due to developmental or functional constraints or homogenous selection (but see [63]). Moreover, since selective pressures cannot be entirely concerted between species, the concerted pattern of brain evolution seen in a cerebrotype hides a host of mosaic adaptations at the species level that further adapts species to their ecology (see [64] for an example). In all cases, the effect of system specific variations highlighted by [9,11] appears to be limited inside cerebrotypes. In heterogeneous taxa however, the diversity of brain composition results from the absence of a shared direction of selection of the brain structure sizes (see also [12]). Thus, the inter-cerebrotypes differences in the scaling of all the brain structures presented here, as well as the presence of grade shifts between taxa [1,9], and that at all levels of the brain betray the differences in the selection of brain structures between cerebrotypes and between species. Therefore, one of the most important issues now is to determine the factors underlying the evolution of each structure, and in particular the reasons why the neocortex and the cerebellum are the structures preferentially enlarged in most, if not all, mammalian cerebrotypes. As developed above, the cerebrotype approach suggests that these two structures are continually under selection, instead of being passively enlarged because of overwhelming developmental constraints. 

#### 3.2.3. The Cerebrotype Approach

Returning to the question raised in the first paragraph, the taxon cerebrotype approach developed here could be of particular value in brain evolution studies by constituting the optimal level of analysis for evo-devo and neuroecological studies. First, the concerted evolution of brain inside cerebrotypes suggests that the number of factors underlying the changes in developmental mechanisms between species should be limited. At this level, the relative and combined effects of developmental and functional constraints and of selective pressures could be studied using the allometric relationships between variables. Second, because selective pressures inside a cerebrotype should be predictable, taxon cerebrotypes could be particularly useful in neuroecological studies. In particular, deviations from the cerebrotype allometry could be more easily understood and would be more relevant than similar analyses in mixed taxa (ecological or taxonomical groups of species). Third, once the mechanisms underlying brain composition inside cerebrotypes would have been determined, it will be possible to describe each cerebrotype with its allometric, size-free parameters. This will enable an in depth understanding of the developmental mechanisms responsible for their differences. The cerebrotype approach is also relatively flexible. In particular, it allows the study of heterogeneous taxa by removing the species with the largest deviations (assuming that the other species do represent a homogenous trend). Also, it permits to study only a limited number of species throughout the range of the cerebrotype, and then predict the others. Therefore, taxon cerebrotype could represent an adequate level of analysis for evo-devo studies and, more generally, for many studies related to brain evolution.

## 4. Methods

### 4.1. Data

The data used is essentially the same as that used by previous authors, *i.e*., from the Stephan, Baron and Frahm’s group [23,24] but with the addition of unpublished data of bat species from the same research group, and measurements of 29 species given in Reep *et al*. [10]. The final dataset consists of the volume of 12 structures (medulla, cerebellum, mesencephalon, diencephalon, striatum, septum, amygdala, paleocortex, hippocampus, schizocortex, neocortex, olfactory bulb) from 376 mammalian species, 28 “insectivores”, 45 primates, 3 scandentia, 271 chiroptera from Stephan Baron and Frahm’s group and 18 carnivores, 5 artiodactyla, 1 perrisodactyla, 1 sirenia and 4 xenarthra from Reep *et al*.’s dataset [10]. The existence of a true “insectivore” taxon is not supported by recent results [28], but is used here for comparison with other studies. In all the analyses, the amygdala is included in the paleocortex as in Stephan *et al*.’s dataset (see [10]). Also, in Reep *et al*.’s [10] measurements, globus pallidus is included in striatum and not in diencephalon as in Stephan *et al.*’s dataset [10].

### 4.2. Analyses

All calculations have been done using R software [65]. A significant phylogenetic signal (K statistic [66]) exists in most taxa and for most variables (using various methods: absolute values, log and proportions, results not shown here). This suggests that phylogenetically based statistical methods are required. In fact, an analysis of the evolutionary changes of brain structure sizes emphasizes taxa specific particularities in how the structures have evolved in time. Analyzing these taxa specific patterns of evolution would provide great insight onto the evolutionary factors at the origin of diversity in brain composition between and inside these taxa. For the purpose of simplification here, a common approach assuming a Brownian model of evolution has been adopted (phylogenetically independent contrasts (PIC) [67]). It is worth noting, however, that more detailed phylogenetic analyses would undoubtedly improve our understanding of brain structures evolution (see [20] for an example with Cichlids). Two phylogenetic trees have been used in this paper. For all taxa except for primates, the “bestdates” supertree of Bininda-Emonds *et al*. [68] was used. Although this phylogenetic tree contains numerous soft polytomies, it offers the possibility to match most of the species of this broad dataset. When calculations were carried out on primates (simians and prosimians), the corresponding tree of the 10ktrees project [69] was used. Because all the species do not match with the mammal supertree, neither with the primate tree, the number of species available after taking into account the phylogeny differs from the original number of species in some taxa. Since the detailed analysis of every taxa is beyond the scope of this study, it was not considered as a problem here. The assumptions of the PIC method have been checked at the taxon level. Although taking a common approach for all the taxa necessarily affects the precision of the statistical analysis, the PIC method should be sufficiently robust here for analyzing the global patterns of brain evolution (and see below).

Only taxa for which more than 15 PIC were available have been included in PIC analysis (10 taxa). The procedure used in Table 1 is described here. First, for each taxon, independent contrasts of the logarithm of each structure were calculated. Second, a Major Axis Regression (MA regression [70]) forced to the origin [71] was carried out for each taxa on each structure regressed on the brain core (as defined by [5], *i.e*., the sum of the medulla, mesencephalon, diencephalon, and striatum). Finally, a test for common slope between groups has been done. Briefly, the *p*-value of the test is obtained by considering that the (Bartlett-corrected) likelihood ratio statistic testing for common slope between groups has a chi-square distribution. The degree of freedom corresponds to the number of groups minus 1 (for details see [70,72]). MA regression was used because in this case, one cannot assume that the size of the brain core predicts the size of other structures. To test whether the results were sensitive to the regression method used, slopes have been calculated with Ordinary Least Square Regression (OLS regression) and then tested with anova following [73]. Although the results are more conservative when OLS regressions are used, the conclusions remain unchanged (results not shown here). Also, the results of the present study are robust to the use of other phylogenetic methods like PGLS and phylogenetic Reduced Major Axis regression (results not shown here).

Phylogenetic Principal Component Analyses (PPCA) [74] have been conducted after calculating the absolute proportions of the brain structures. The same methodology as [15] has been used, *i.e*., dividing the size of each structure by the size of the entire brain. The olfactory bulb is not included in the carnivores’ taxon because it is not included in [10]. It is likely however that further analysis with olfactory bulb would have different conclusions (a lower homogeneity of the species) [10]. Correlations between each principal component (PC) and absolute brain size have been tested by using OLS regression through the origin, of the PIC of the position of each species on each PC onto the PIC of absolute brain size. More sophisticated phylogenetic methods could undoubtedly precise the relative position of the species between each other, but they are unlikely to fundamentally change the observed patterns. Indeed, the same patterns (presence/absence and strength of a tendency for brain composition to vary with brain size) are found when using classical, non-phylogenetic principal component analyses (results not shown here). The diameter of the discs in the 3D PPCA indicates the relative size of the brains in the taxon, *i.e*., the species with the biggest brain in the taxon possesses the largest disc, and the area of the disc of all the other species is proportional to the volume of their brains compared to the volume of the biggest brain in the taxon.

## 5. Conclusions

Accumulating evidences suggest that constraints (developmental and functional) have played an important role in mammalian brain evolution. Despite this apparent limitation, various mechanisms have been selected during mammalian evolution to produce the diversity of cerebrotypes. By studying a growing number of individuals, species and taxon cerebrotypes, further studies should determine the degree to which each factor; genetic, developmental and functional are involved in brain evolution and its behavioral outcome. For example, one promising field of interest is the study of genetics; in particular, conditions like microcephaly or primordial dwarfism for which there are extreme variations of brain size with or without other modifications [75]. Genes involved in microcephaly are thought to play a role in brain size evolution in primates [76], and particularly in humans [77], although the mechanisms at the origin of the microcephaly are still largely unknown [75]. It remains for future work to clarify how genes known to be involved in these conditions are involved in the evolution of brain composition inside a cerebrotype, and not only in absolute brain size variation. As illustrated here by the question of modularity and constraints in brain structure evolution, the cerebrotype approach could be a valuable tool for our understanding of brain evolution. 

## Supplementary Files

Supplementary File 1 PDF-Document (PDF, 491 KB)
