# Supplementary material for: Understanding the Evolution of Mammalian Brain Structures; the Need for a (New) Cerebrotype Approach
Source: Brain Sci. 2012 May 18;2(2):203–24. doi: 10.3390/brainsci2020203 (PMC4061787; doi:10.3390/brainsci2020203)
Supplement: Supplementary File 1 — PDF-Document (PDF, 491 KB) [file brainsci-02-00203-s001.pdf]

# Supplementary Information

**Figure S1.** Phylogenetic Principal Component Analysis of Hipposideridaes' brain structure proportions. Regression on independent contrasts of logged absolute brain size onto independent contrasts of position on PC1: Multiple R-squared: 0.621, F-statistic: 29.54 on 1 and 18 DF, $P = 3.654 \times 10^{-5}$; PC2: Multiple R-squared: 0.173, F-statistic: 3.771 on 1 and 18 DF, $P = 0.06795$.

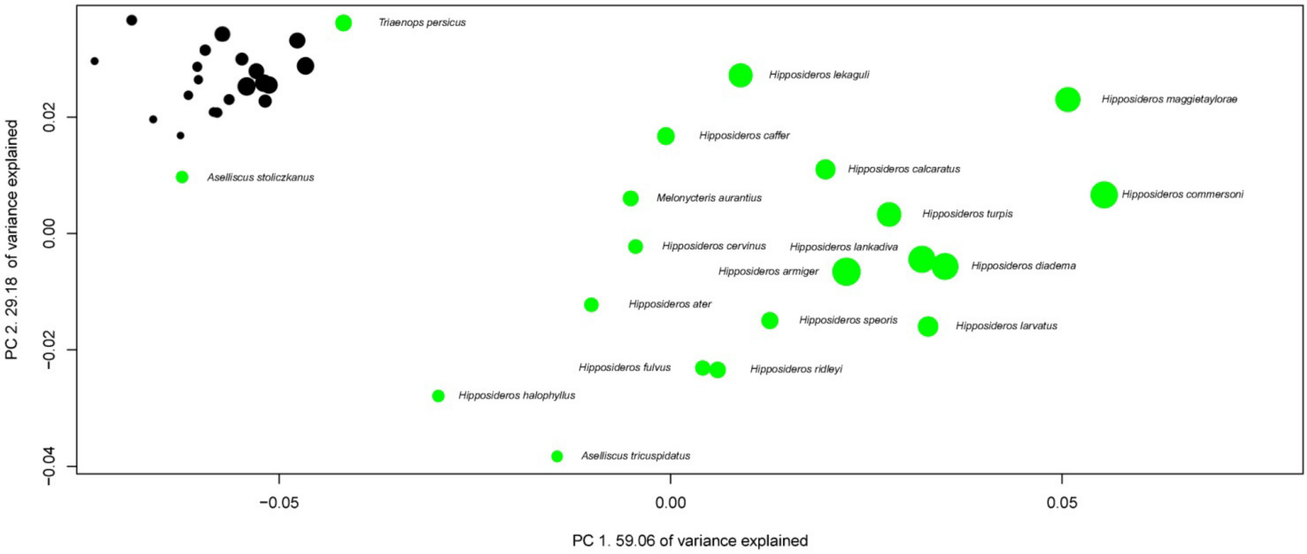


**Figure S2.** Phylogenetic Principal Component Analysis of Molossidaes' brain structure proportions. Regression on independent contrasts of logged absolute brain size onto independent contrasts of position on PC1: Multiple R-squared: 0.0221, F-statistic: 0.3384 on 1 and 15 DF, $P = 0.5694$; PC2: Multiple R-squared: 0.705, F-statistic: 35.86 on 1 and 15 DF, $P = 2.486 \times 10^{-5}$.

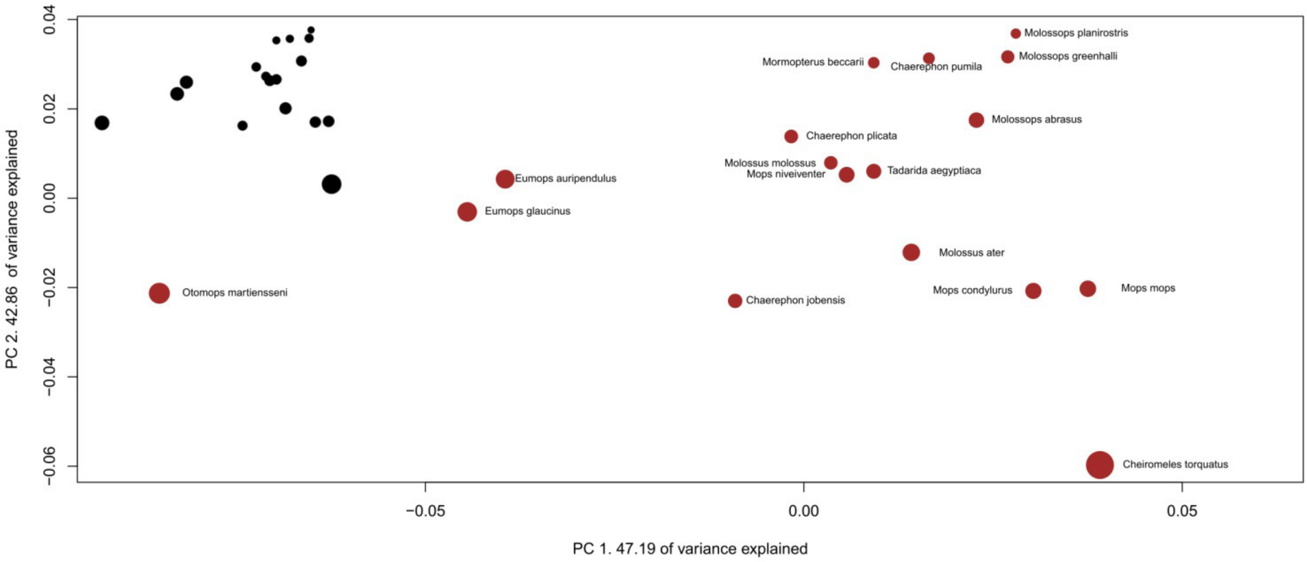

**Figure S3.** Phylogenetic Principal Component Analysis of Emballonuridaes' brain structure proportions. Regression on independent contrasts of logged absolute brain size onto independent contrasts of position on PC1: Multiple R-squared: 0.0095, F-statistic: 0.1437 on 1 and 15 DF, $P = 0.71$; PC2: Multiple R-squared: 0.4503, F-statistic: 12.29 on 1 and 15 DF, $P = 0.003189$.

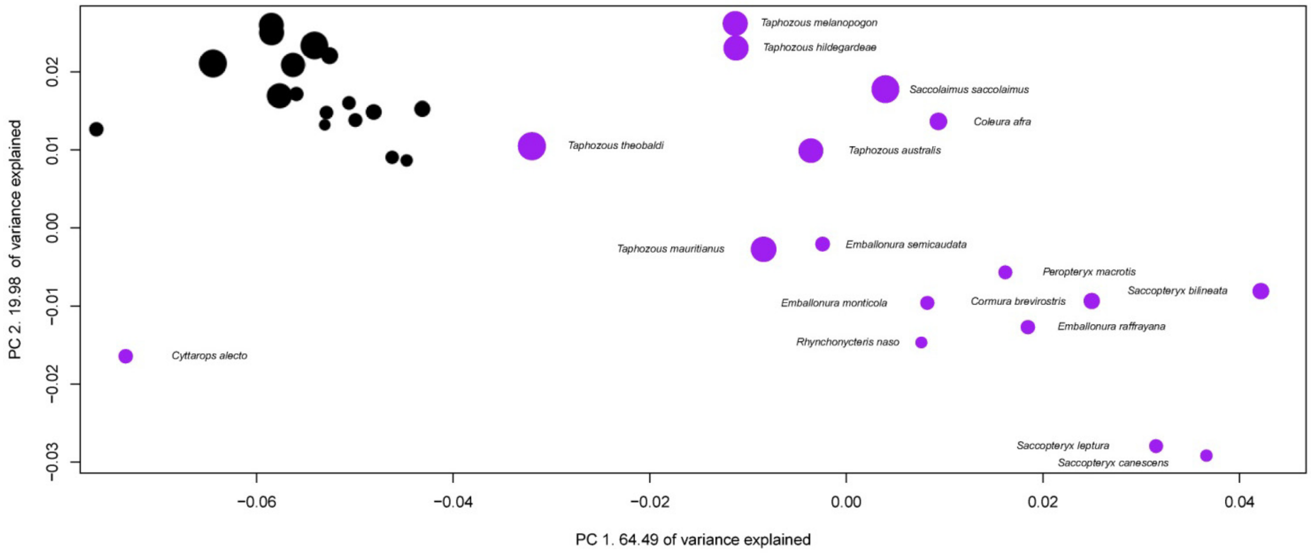


**Figure S4.** Phylogenetic Principal Component Analysis of Phyllostomidaes' brain structure proportions. Regression on independent contrasts of logged absolute brain size onto independent contrasts of position on PC1: Multiple R-squared: 0.0770, F-statistic: 4.006 on 1 and 48 DF, $P = 0.051$; PC2: Multiple R-squared: 0.1223, F-statistic: 6.69 on 1 and 48 DF, $P = 0.01278$.

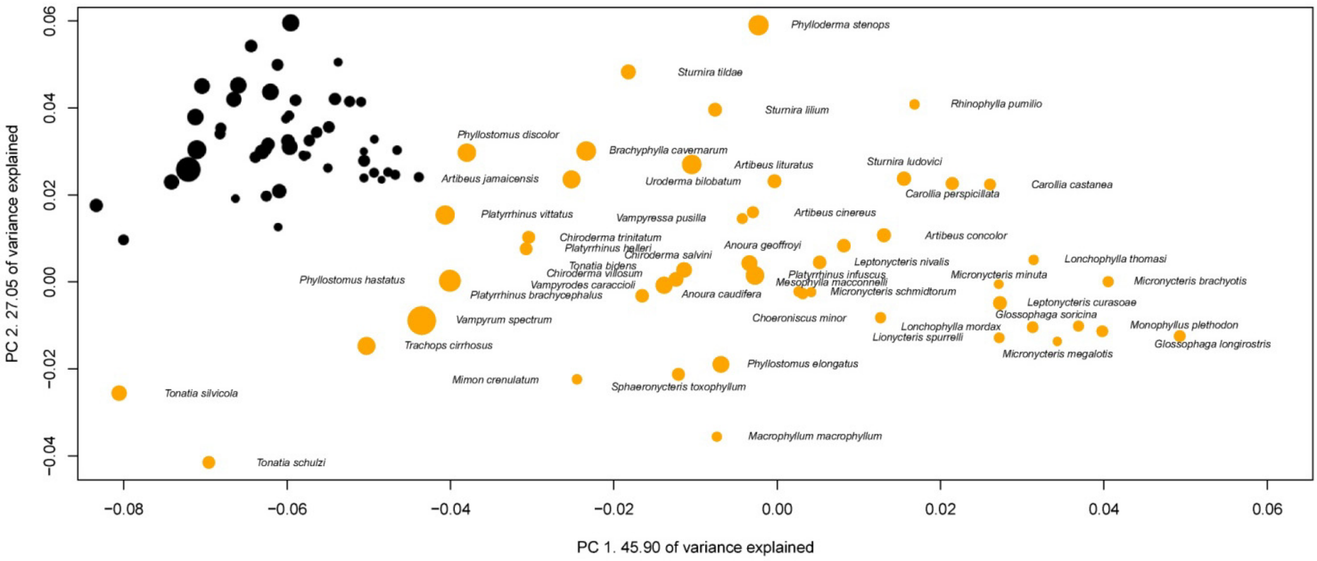

**Table S1.** Loadings of the brain structures for the five first principal components of PPCA for each taxon of the Figures S1 to S4. Scores higher than 0.5 are highlighted in grey.

| | **PC 1** | **PC 2** | **PC 3** | **PC 4** | **PC 5** | | **PC 1** | **PC 2** | **PC 3** | **PC 4** | **PC 5** | |
|---|---|---|---|---|---|---|---|---|---|---|---|---|
| **Phyllostomidae** | **45.897** | **27.050** | **15.118** | **5.943** | **1.960** | **% variance** | **47.191** | **42.858** | **4.430** | **1.749** | **1.416** | **Molossidae** |
| | 0.138 | −0.834 | −0.462 | 0.008 | −0.160 | **Medulla** | 0.203 | 0.906 | 0.247 | −0.194 | −0.049 | |
| | 0.308 | −0.492 | 0.809 | 0.086 | −0.012 | **Cerebellum** | −0.997 | −0.074 | 0.011 | 0.016 | −0.001 | |
| | 0.045 | −0.792 | −0.442 | 0.155 | 0.351 | **Mesencephalon** | 0.238 | 0.805 | 0.467 | 0.152 | 0.054 | |
| | −0.219 | −0.356 | −0.463 | −0.003 | −0.280 | **Diencephalon** | 0.720 | 0.097 | −0.069 | 0.062 | 0.551 | |
| | −0.176 | 0.399 | −0.210 | −0.026 | −0.506 | **Striatum** | 0.411 | −0.365 | −0.493 | 0.103 | 0.479 | |
| | 0.409 | 0.514 | −0.236 | −0.325 | −0.117 | **Septum** | 0.428 | 0.286 | −0.631 | −0.121 | 0.126 | |
| | 0.483 | 0.740 | −0.148 | 0.394 | −0.040 | **Paleocortex** | 0.611 | 0.408 | −0.119 | 0.531 | −0.337 | |
| | 0.661 | 0.363 | 0.025 | −0.635 | 0.096 | **Hippocampus** | 0.510 | 0.438 | −0.571 | −0.215 | −0.240 | |
| | 0.210 | 0.556 | 0.051 | −0.511 | 0.028 | **Schizocortex** | −0.089 | 0.236 | 0.026 | −0.679 | −0.268 | |
| | −0.991 | 0.092 | 0.083 | −0.018 | 0.021 | **Neocortex** | 0.226 | −0.964 | 0.135 | −0.016 | −0.027 | |
| | 0.412 | 0.798 | 0.057 | 0.304 | 0.129 | **Olfactory bulb** | 0.498 | −0.187 | −0.691 | 0.013 | −0.043 | |
| | **PC 1** | **PC 2** | **PC 3** | **PC 4** | **PC 5** | | **PC 1** | **PC 2** | **PC 3** | **PC 4** | **PC 5** | |
| **Emballonuridae** | **64.493** | **19.982** | **6.350** | **4.209** | **1.994** | **% variance** | **59.057** | **29.180** | **4.883** | **3.152** | **2.435** | **Hipposideridae** |
| | −0.321 | −0.485 | 0.743 | −0.323 | 0.030 | **Medulla** | −0.788 | −0.453 | 0.348 | 0.211 | 0.065 | |
| | −0.993 | 0.080 | −0.036 | 0.065 | 0.031 | **Cerebellum** | −0.669 | 0.742 | −0.008 | −0.034 | 0.021 | |
| | 0.223 | −0.920 | −0.305 | −0.024 | 0.041 | **Mesencephalon** | −0.530 | −0.643 | 0.210 | −0.503 | −0.029 | |
| | 0.399 | −0.587 | −0.458 | −0.039 | 0.220 | **Diencephalon** | 0.506 | −0.677 | −0.206 | −0.261 | 0.124 | |
| | 0.339 | 0.239 | 0.234 | 0.255 | −0.230 | **Striatum** | 0.512 | −0.388 | −0.577 | −0.215 | 0.307 | |
| | 0.639 | −0.009 | 0.172 | 0.260 | −0.344 | **Septum** | 0.124 | −0.714 | −0.330 | −0.171 | −0.188 | |
| | 0.902 | 0.271 | 0.119 | 0.238 | 0.187 | **Paleocortex** | 0.721 | −0.475 | −0.335 | 0.147 | 0.292 | |
| | 0.506 | −0.204 | 0.104 | −0.108 | −0.693 | **Hippocampus** | −0.565 | −0.353 | −0.453 | 0.167 | −0.551 | |
| | 0.521 | 0.376 | −0.065 | 0.510 | −0.426 | **Schizocortex** | 0.172 | −0.200 | 0.059 | 0.444 | −0.605 | |
| | 0.283 | 0.785 | −0.313 | −0.451 | −0.003 | **Neocortex** | 0.933 | 0.310 | 0.171 | −0.017 | −0.057 | |
| | 0.203 | 0.650 | 0.141 | −0.177 | 0.363 | **Olfactory bulb** | 0.760 | 0.263 | −0.157 | 0.299 | 0.371 | |
